# Towards a public health approach for palliative care: an action-research study focused on engaging a local community and educating teenagers

**DOI:** 10.1186/s12904-018-0344-y

**Published:** 2018-06-29

**Authors:** Sandra Martins Pereira, Joana Araújo, Pablo Hernández-Marrero

**Affiliations:** 1 Instituto de Bioética, Universidade Católica Portuguesa, Rua Diogo Botelho, 1327 4169-005 Porto, Portugal; 2 UNESCO Chair in Bioethics Instituto de Bioética, Universidade Católica Portuguesa, Porto, Portugal; 3 CEGE: Centro de Estudos em Gestão e Economia [Research Centre in Management and Economics Católica Porto Business School, Universidade Católica Portuguesa, Porto, Portugal

**Keywords:** Public health education, Palliative care, Teenagers, Action research, Compassionate communities, Community-based intervention, Public health policy

## Abstract

**Background:**

Education sessions about palliative care among teenagers are uncommon in developed countries. However, very little is known either about the impact of this type of intervention or about how this age-group perceives its impact. The purpose of this study was therefore to (i) implement an education program about palliative care among teenagers and (ii) to investigate the impact of the program on the participants.

**Methods:**

An action-research study was conducted at a local community parish in Portugal in November 2015. An education programme was purposively built about palliative care, using active educational strategies adapted for teenagers. Quantitative and qualitative techniques and instruments were used for data collection: questionnaire; reflective diaries; interviews and written testimony. The program had three stages: preparation; intervention; and evaluation. Qualitative data were analysed using thematic content analysis; quantitative data were analysed descriptively.

**Results:**

69 people (47 teenagers) participated in the education program. Findings show that the education program contributed to creating awareness about palliative care. Both the teenagers and other participants assessed the education program positively. At the end of the program, teenagers had a constructive message about palliative care.

**Conclusions:**

The education-intervention contributed to create awareness about palliative care among the participant teenagers, who ended the program with a positive message about palliative care. Based on our findings, the following policy implications can be drawn: (1) Further research is needed to evaluate the effect of education programs about palliative care among younger age groups (teenagers and children), particularly in relation to the changing of attitudes toward palliative care. (2) Education about palliative care should be promoted to local communities, involving all age groups, to foster involvement, participation and empowerment. (3) Compassionate communities should be promoted to enhance the health and wellbeing of all citizens at the end of their life.

## Background

Healthcare education is paramount to ensure citizens’ awareness, knowledge and empowerment on any issues related to health. It involves giving information and teaching individuals and communities about how to achieve better health [1–3].

The worldwide trend of ageing populations raises major challenges to health (e.g., increasing number of older people with several co-morbidities, chronic, progressive diseases, among others). Health promotion activities through education become therefore increasingly relevant. Health issues need to be addressed by using a holistic approach that fosters individuals and communities’ empowerment to take action both for their health and for the development of inter-sectoral actions to build public policies capable to create and maintain sustainable healthcare systems [1–4]. In this context, palliative care, defined as ‘an approach that improves the quality of life of patients and their families facing the problems associated with life-threatening illness, through the prevention and relief of suffering by means of early identification and treatment of other problems, physical, psychosocial and spiritual’ [5], has progressively been recognized as an essential ethical responsibility of health systems worldwide [6–11].

Evidence shows that public awareness of the concept of palliative care remains insufficient [12–14], thus showing the need to further implement societal actions and education programs on this matter. This has been identified worldwide as one of the key pillars of a public health strategy for palliative care [15–17].

Health-promoting palliative care activities are aimed at implementing education and information programs, reorienting health education and services toward community partnerships [2, 3, 18]. This may be particularly helpful to develop more compassionate communities, defined as communities that understand that contributing to the care of those living with a life-threatening disease is an intrinsic part of the health and well-being responsibilities of all citizens [18, 20].

To be successful and effective, healthcare education strategies and programs, especially those focusing on sensitive issues like palliative and end-of-life care, should be participatory and involve the wider community [18–20]. Involving schools, workplaces, places of worship, the media or local businesses can help mobilize untapped sources of care and support as well as practical resources [20].

As the health impact of education lasts a lifetime [21–24], it is paramount that healthcare education programs begin during the early stages of human development. These programs should be culturally and educationally appropriate [25] and can foster the development of critical perspectives towards issues and problems with implications for human health [24].

Education activities about palliative care (e.g., short thematic sessions) among teenagers are uncommon practices in developed countries. Therefore, very little is known about how this age group perceives the impact of this type of interventions. Despite a few initiatives, there is limited research available on the process of implementing educational programs about palliative care specifically focused on teenagers. To our best knowledge, only two studies have been developed on this matter [25, 26]. These two pilot-studies aimed at developing and piloting an in-depth intervention on severe illness and palliative care in high schools, assessing students’ interest in and knowledge of palliative care, the overall impact of this experience, and the usefulness of the intervention components and procedures in both teachers and students [25, 26]. The findings of these studies suggest that teenage high school students deemed the education program about palliative care to be a helpful and positive experience [25, 26].

Triggered by the abovementioned experiences, the objectives of our study were: (i) to implement an education program about palliative care among teenagers and (ii) to investigate the impact of the program on the participants.

## Methods

An action-research study was conducted in a local community parish in the North of Portugal. A mixed-methods approach was used for data collection, combining both quantitative and qualitative techniques and instruments to achieve more comprehensiveness. The triangulation or synthesis of multiple sources of data is a core element of action-research as it is very important to ensure that all available data is used to build rigorous and cohesive conclusions [27, 28].

Action-research seeks to bring together action (the education-intervention program) and reflection (how to make this education-program effective and positive, and how to create knowledge about palliative care among teenagers), theory and practice, in participation with others (teenagers, parents, catechists – i.e., people responsible for the religious education activities at a parish, the community, the educator-researcher) in the pursuit of practical solutions to issues of pressing concern to people [29–31]. This methodological approach is of special relevance in palliative care and education [31–34], particularly in terms of community-based interventions aimed at improving change [34–37]. Following the action-research approach, the education program was tailored to the specific context of its implementation [38].

As a research methodology, action-research focuses on how people’s situations can be improved and helps to empower people through the process of constructing and using their own knowledge [30, 39, 40]. Furthermore, action-research helps to transform organizations and/or communities into collaborative and self-reflective contexts, empowering subjects (researchers and participants) as part of the process [39, 40].

The education program and action-research assumed the form of the typical cycle or spiral of action-research [31, 34, 35, 40] and embraced three intertwined stages:

1. a preparatory stage, during which the participating teenagers were invited to write a sentence and/or question about palliative care;
2. the education-intervention stage, i.e., an education session rooted and developed using the sentences/questions written by the teenagers during the preparatory stage;
3. the evaluation stage, during which the participants assessed the overall education program using a questionnaire entailing a set of four dimensions: Organization of the overall education program, Preparatory stage, Education-intervention stage (session), and Evaluation stage (possibility to assess the education program), which were scored on a seven-point Likert scale ranging from 1 = very bad to 7 = very good. This evaluation tool also included two open questions entitled “Additional comments” and “Take-home message” resulting from the education-intervention stage.

All stages were guided and followed by the researcher-educator together with the catechist responsible for the religious education activities of the group of teenagers at the participant parish. It is worth mention that the initial contact was made by the catechist of the parish who approached the researcher-educator with the idea of implementing this type of initiative. The study was conceived as a way of comprehending the impact of the education program for teenagers among the participants. The education program on palliative care for teenagers was structured as follows:

1. Preparatory session:

The preparation session was held approximately one month before the education session. This session was led by the catechist and had a duration of 2 h. The following contents and activities were held during this session:

Concepts of palliative and end of life care.

Brainstorming of ideas and questions on palliative and end of life care.

2. Education session:

The education session was based on the ideas and questions raised by the teenagers during the preparatory session. This session was led by a palliative care nurse and had a duration of 3 h. The following contents were taught using an active and interactive approach:

Clarification of concepts: palliative and end of life care.

Answer to the ‘what, why, who, when, where, how’ questions in relation to palliative and end of life care.

Ethical and existential issues in palliative and end of life care.

3. Evaluation:

No specific session was designed for the evaluation. The program was evaluated throughout its duration, following the principles and approaches of action-research.

This catechist responsible for the group of teenagers had a relevant role in the implementation of this initiative. The latter was fully supported by the reverend who decided to open the education session to the local community. This is aligned with the principles and practices of action-research where the stakeholder participants have an active role in decision-making [31].

The education program and data collection period occurred from October 2015 to December 2015. The education session happened on November 6, 2015, and had a duration of three hours. Interviews were held in the weeks following the education session. Further details on the data collection are described in the next section.

### Instruments for data collection

As aforementioned, a mixed-methods approach was used for data collection as this brings more comprehensiveness and is coherent with action-research. The following data were collected: (1) the complete list of sentences/questions written by the teenagers during the preparatory stage; (2) the field-notes written by the researcher-educator during the overall duration of the education program; (3) the evaluation questionnaire applied to the participants who attended the education session; (4) semi-structured interviews conducted with the catechist responsible for the group of teenagers and two community members (adults) who participated in the education session; and (5) a written testimony about the session written by the reverend at his own initiative and publicly available.

The interview guide for the semi-structured interviews included the following questions:

1. Could you please share your thoughts about organizing/attending this education program/session about palliative and end of life care?
2. Do you think that the program was well tailored for teenagers?
3. Considering the three stages of the education program (i.e., the preparatory stage, the education session, and the evaluation stage), how would you evaluate each one of them?
4. What were the main weaknesses and strength of this initiative?
5. Would you repeat this experience? If so, what suggestions do you have for improvement?
6. Is there something else that you would like to add or share about this initiative?

The combination of diverse instruments for data collection is an inherent dimension of action-research. It is worth mention that the inclusion of field-notes (also named as reflective diaries) and written testimonies is a common component of palliative and end-of-life care education programs, and of education research, as they allow participants and researchers-educators to revisit their own experience [41].

**Fig. 1:**
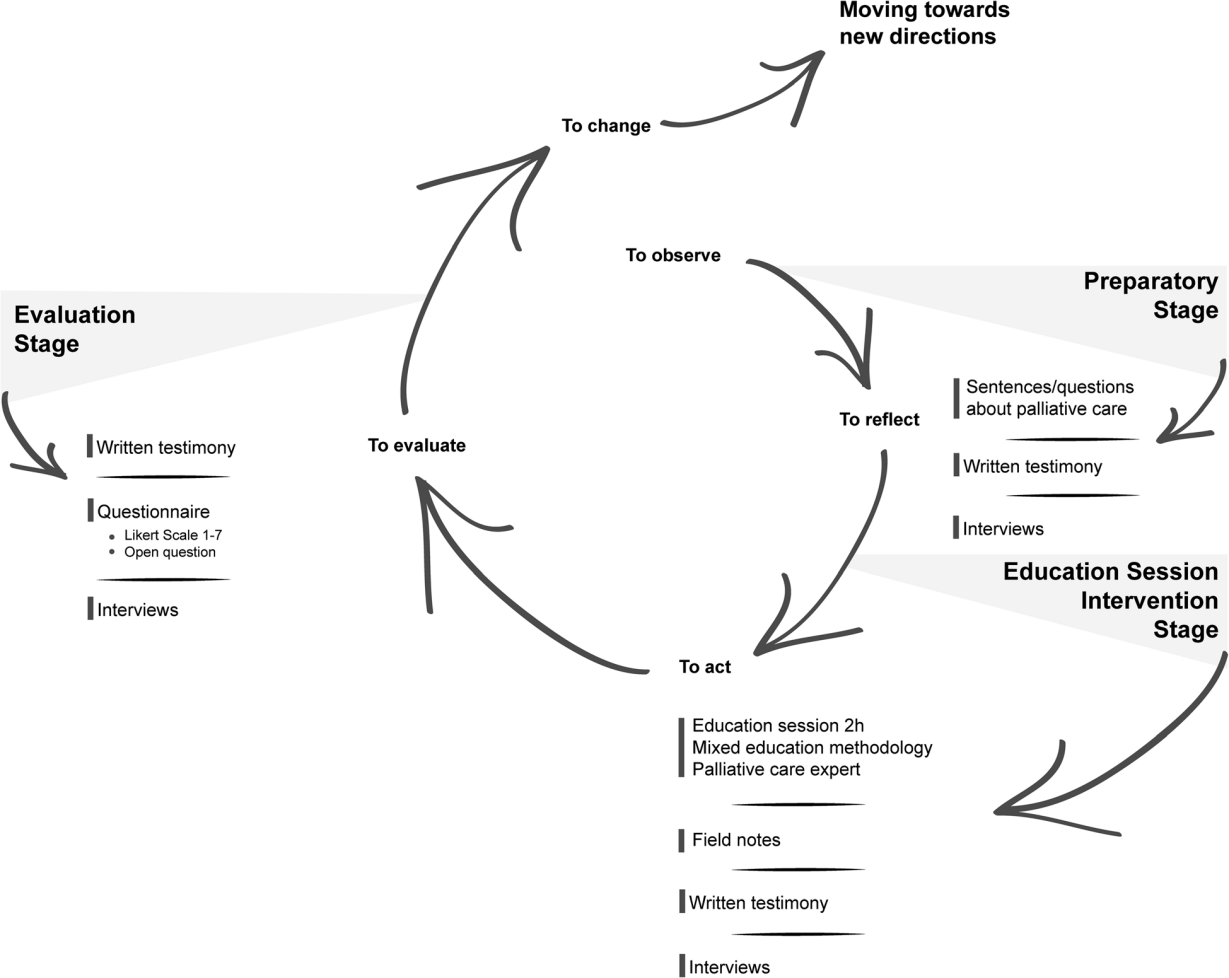
The action-research spiral combining and integrating the techniques and instruments used for data collection

Figure 1 illustrates the combination and integration of techniques and instruments used for data collection within the action-research spiral.

### Participants, data collection and ethics approval

The education-program and research-action on palliative care for teenagers was held at a community parish in the North of Portugal. Participants were a convenience sample of teenagers attending the religious education activities of this parish, the catechists and community members.

A total of 69 persons participated in the education session, 67 (97%) of whom completed the evaluation questionnaire. Although specifically prepared for teenagers (69% of the participants), the education session also included the participation of 8 parents (12% of the participants), 11 catechists (16% of the participants) and 2 members of the local community parish (3% of the participants). The teenagers were aged between 12 and 18 (72% aged between 15 and 18), with the majority being female. The educator-researcher had a clinical background as a nurse and more than ten years of experience in the field of palliative care.

Ethical approval was obtained from the Ethics Lab: Ethical Analysis, Consultation and Monitoring of Scientific Research Projects and Clinical Trials (former SACE: Serviço de Análise e Consultoria Ética de Projetos de Investigação Científica e Ensaios Clínicos) of the Universidade Católica Portuguesa [Ref.08.2015]. As the study involved minors (i.e., the participant teenagers), a few specific ethical procedures were followed and ensured, namely: (i) Parents and other educators were involved sidelong the education and research process; (ii) parents were informed and consented teenagers to be engaged in the education-research process about palliative care; (iii) verbal parental consent was acquired for participants under 18 years of age for participation in the study; (iv) no confidential data were collected; (v) anonymity was fully ensured; and (vi) no harm was caused to any of the participants. In fact, due to the sensitivity of the topic, researchers and educators were informed upfront if any teenagers were experiencing the process of a life-threatening disease (personally or through a next-to-kin); there was no such situation among the participants. In addition, all data were anonymized, analysed and presented with maximum confidentiality.

### Analysis

#### Data analysis varied as follows:

1. Qualitative data: Both content and thematic analysis were applied to the sentences and questions written by the teenagers during the preparatory stage, to the field-notes written by the researcher-educator, to the answers given by the participants to the open questions of the questionnaire (i.e., “Additional comments” and “Take-home messages”), to the transcripts of interviews, and to the written testimony. This approach allowed the analysis of multifaceted, important and sensitive phenomena of educating teenagers on palliative and end of life care [42].
2. Quantitative data: A descriptive analysis was performed on the questions of the evaluation questionnaire. Percentages were calculated for each dimension and item.

Data were analysed independently by two researchers (SMP and PHM).

The triangulation steaming from the use of different instruments, data and researchers ensures the reliability and comprehensiveness of the findings. This type of approach is useful to build meaning from different sources [43, 44]. This is a valuable strategy used in qualitative research, such as this form of action-research, as engaging multiple methods and techniques leads to more valid, reliable and diverse construction of realities [43–49].

Table 1 shows the integration of the education program and research methods.

**Table 1:** Integration of the education programme and research methods

Education programme on palliative care for teenagers	Participants	Method for data collection	Analysis
Preparatory stage	Teenagers (*n* = 48) Catechists (*n* = 11)	Written sentences/ questions about palliative care	Thematic content analysis
		Interviews	
	Reverend	Written testimony	
	Researcher-educator	Field notes	
Intervention stage ‘Education Session’	Teenagers (n = 48) Parents (*n* = 8) Catechists (n = 11) Community members (*n* = 2)	Education session materials (PPT)	Thematic content analysis
		Interviews	
	Reverend	Written testimony	
	Researcher-educator	Field-notes	
Evaluation stage	Teenagers (n = 48) Parents (n = 8) Catechists (n = 11) Community members (n = 2)	Questionnaire	Descriptive (%)
		Interviews	Thematic content analysis
	Reverend	Written testimony	
	Researcher-educator	Field-notes	

Note: Although the evaluation stage is described at the end of the process, it focused on all stages of the education programme

## Results

Results are presented considering the education program on palliative care. Data and information gathered from the different sources are integrated to provide a more comprehensive answer to our research objectives, namely: to implement and understand the impact of an education program about palliative care for teenagers.

### The preparatory stage

During the preparatory stage, the teenagers solely wrote questions about palliative care. These questions were organized into eight domains: Definitions of concepts; Articulation (palliative versus curative care); Care beneficiaries; Care timings and timeframe; Organization; Directories; Economic costs; Philosophy, practice and meaning(s) (Table 2).

**Table 2:** Themes and categories emerging from the preparatory stage

Dimensions	Categories	Examples of text units
Definition of concepts	–	*“What is palliative care?”* (WQS12)
Articulation	Palliative care versus curative care	*“Does palliative care prevent patients from receiving medical drugs or treatments?”* (WQS2)
Care beneficiaries	–	*“Does palliative care act on patients in vegetative condition?”* (WQS 4)
Care timings and timeframe	–	*“Are patients in palliative care cared by these teams until death or is there a timeframe?”* (WQS 3)
Organization	–	*“How is a palliative care team compounded?”* (WQS 1)
Directories	–	*“How is it possible to find palliative care resources?”* (WQS13)
Economic costs	–	*“(…) is palliative care cost-free for patients?”* (WQS 6)
Philosophy, practice and meaning(s)	Philosophy and ethics	*“Do we have the right to end the life of a person who is between life and death?”* (WQS11)
	Meaning(s)	*“Is there happiness (…) in palliative care?”* (WQS10)
Relevance and depth of questions	–	*“The questions raised by the teenagers are very interesting, relevant and show some in-depth reflection about palliative care”* (RFN1)
Role of the community catechist	–	*“Mr. X, catechist of our community parish, was the inspirer of this education initiative…”* (WT)

*WQS* Written Questions/Sentences by teenagers. *RFN* Researcher Field Notes. *WT* Written Testimony

In the written testimony, the reverend valued the fact that this education-intervention program resulted from the initiative of a community catechist, who organized the overall action and had a major role during its preparatory stage. According to the educator-researcher’s field-notes and one of the interviewees (the catechist in charge of the group of teenagers), the questions raised by the teenagers were of relevance and depth. Table 3 illustrates themes and categories emerging from the education-intervention stage.

**Table 3:** Themes and categories emerging from the education-intervention stage

Categories	Examples of text units
Openness and positive attitude of the teenagers	*“(…) teenagers were very attentive, paying attention to the answers given to the questions they raised during the preparatory stage… They also looked very open, interested and willing to learn more about palliative care”* (RFN) *“I must acknowledge the clear interest shown by all participants, especially our teenagers, to this bioethical dialogue”* (WT)
Teenagers’ high participation during the education-intervention session	*“(…) teenagers wanted to participate more…”* (RFN) *“It was quite positive to see how many questions the teenagers raised during the session”* (RFN)
Positive feature of opening the session to the parents and local community	*“It is really important and positive that parents and other members from the local community attended the education session!”* (RFN) *“I am very grateful that our priest wanted to open this session for anybody willing to attend it. I feel now more confident and prepared to further discuss these issues with my family and friends”* (I2)

*RFN* Researcher Field Notes. *WT* Written Testimony. *I* Interviewee

### The education-intervention stage

The education-intervention stage had a three-hour duration and comprised a mixed-strategy. This session was fully developed based on the questions raised by the teenagers and interleaved some more theoretical moments with interactive ones. Field-notes revealed the following dimensions: Openness and positive attitude of the teenagers; teenagers’ high participation during the education-intervention session; and positive feature of opening the session to the parents and local community. In his written testimony, the reverend also valued the positive attitude of the teenagers towards the education-intervention about palliative care. The two community members who were interviewed also acknowledged the possibility of attending the education session, as it allowed them to improve their knowledge about palliative care, feeling now more able to further discuss palliative and end of life care issues with other people (Table 2).

### The evaluation stage

The questionnaire allowed the evaluation of the following items: “Organization of the education program”, “Preparatory stage”, “Education-intervention stage/session”, and “Possibility to evaluate the education program”. 71% of the participants rated the “Organization of the education program”, the “Preparatory stage”, and the “Possibility to evaluate the education program” with 7 points (Very Good). The “Education-intervention stage/session” was assessed as being very good by 65% of the attendees. Table 4 shows the complete evaluation rates.

**Table 4:** Evaluation rates of the education session

Stage of the Education Program	Qualitative assessment	Scoring in the Likert Scale	*N* = 67%
Organization of the overall education program	Very Good	7	71%
		6	15%
		5	8%
		4	6%
		3	0%
		2	0%
	Very Bad	1	0%
	Total	–	100%
Preparatory stage	Very Good	7	71%
		6	15%
		5	7%
		4	7%
		3	0%
		2	0%
	Very Bad	1	0%
	Total	–	100%
Education-intervention stage (session)	Very Good	7	65%
		6	22%
		5	5%
		4	8%
		3	0%
		2	0%
	Very Bad	1	0%
	Total	–	100%
Evaluation stage (possibility to assess the education program)	Very Good	7	71%
		6	22%
		5	6%
		4	1%
		3	0%
		2	0%
	Very Bad	1	0%
	Total	–	100%

From the content analysis performed to the answers given to the open questions, two major dimensions emerged, compelling the following categories: Main take-home messages, namely “Moments of happiness” and “Societal relevance”, and Additional comments, explicitly “Excellent”, “Exciting” and “Enlightening”. In addition, the field-notes and transcripts of interviews showed that the duration of the session was short, highlighting the need for more time in order to ensure a proper discussion of all questions and ideas raised by the participant teenagers. Findings from the written testimony also show the gratitude of the community parish and reverend for having had the opportunity to host and participate in this education-intervention program about palliative care (Table 5).

**Table 5:** Themes and categories emerging from the evaluation stage

Dimensions	Categories	Examples of text units
Take-home messages	Moments of happiness	*“Happiness can exist during a bad disease”* (AWT2) *“… nurturing the value of life in order to experience happiness”* (WT)
	Societal relevance	*“Palliative care is crucial for the population”* (AWT48)
Additional comments	Excellent	*“Excellent session!”* (AWE7)
	Exciting	*“Exciting”* (AWT38)
	Enlightening	*“(…) enlightening and with very clear and relevant information”* (AWE66)
	Revolutionary	*“(…) revolutionary education session”* (WT)
	Thank you	*“(…) the gratitude we feel for having had this education programme. Thank you!”* (WT)
Education session: Need for more time		*“it would have been good to have more time to further discuss some of the issues and give a more complete and comprehensive answer to the questions raised by the teenagers”* (RFN) *“We really need more sessions like this one! This one was great, but we needed more time to deepen some discussions.”* (I1)

*AWT* Answers written by teenagers. *AWE* Answers written by catechists. *RFN* Researcher Field Notes. *WT* Written Testimony. *I* Interviewee

## Discussion

Our findings show the positive impact of implementing an education-intervention strategy and program aimed at increasing awareness about palliative care among teenagers. It highlights the openness of both teenagers and their parents to this topic, as well as the relevance of engaging the community in this type of initiatives. This is aligned with the two previous studies identified in our literature review, suggesting that educating teenagers and high school students about palliative care is not only appreciated by them, but also improves their attitudes toward dying, death and loss [25, 26].

As suggested by Becarro et al. [25, 26], our education-intervention program was appreciated by the teenagers. This was shown by the high level of attention and participation during both the preparatory stage and education-intervention session, and by the feedback given during the evaluation stage. The majority of the participant teenagers asked for more time to allow a more thorough discussion of more sensitive topics. This was reinforced by the two community members (adults) who also attended the session.

The in-depth and quality of the questions raised during the preparatory stage show the openness and interest teenagers have about palliative care. Moreover, it suggests the societal impact of education programs on this matter, specifically when targeted and tailored for teenagers. In contrast to traditional and wide-spread sources of health information (e.g., flyers and conventional education program), interactive and interpersonal health communication and health education strategies offer the potential for more individually tailored messages. This may have contributed to the positive evaluation of our action-research program as the participants, particularly the teenagers, had an active role throughout the overall set of activities and phases.

It is worth mention that some of the questions raised by the teenagers focused on ethical issues, such as euthanasia and assisted suicide, equity in the access to palliative care, organizational aspects and integrative care approaches. While a previous study published elsewhere showed that end-of-life decisions were accepted by adolescents under certain circumstances [50], our findings suggest that the participant teenagers were more interested in discussing these ethical issues rather than on having a clear position or attitude towards them. This is in line with a study conducted about teaching bioethics in high schools, which indicated that teenage students were enthusiastic and willing to discuss ethical issues [24]. In our study, this preference to discuss rather than assuming a position may have occurred for two main reasons. On the one hand, our study and education-program was implemented in a religious context. This may have prevented teenagers from assuming publicly their positions and needs further clarification. On the other hand, another possible explanation for the interest in discussing these topics may have been because of the current public and legal debate on the legalization of euthanasia and physician assisted suicide in Portugal.

Another interesting finding of our study was the involvement shown by parents, catechists and other members from the local community. In fact, considering that the education-intervention session was held on a Sunday morning, it is remarkable that about 30% of the participants did not belong to the teenage group who regularly have their meetings on this day. This shows the local potential of this specific community in terms of awareness about palliative care, fostering the potential of the participant teenagers to further discuss some of the topics developed during the education-intervention session. The participation of adult members of the community in the education-intervention session is also a sign of the possibility to develop a compassionate community, more able to contribute to the actual care of those in need of palliative care [19, 20]. Previous studies have shown that community-based educational initiatives promote knowledge and awareness of palliative care [13, 20], which can promote empowerment [2, 3, 36] and foster the implementation of initiatives to improve care provision at the end of life for all citizens. Direct engagement with communities can indeed improve a multicultural understanding of populations’ cultural and health needs about palliative and end of life care [51].

Finally, it is remarkable how the education-session contributed to spread a positive and constructive message about palliative care among the participant teenagers. Based on the “Take-home messages” written by these teenagers, at the end of the education session/program, they perceived palliative care as having the potential to contributing to “moments of happiness” for those suffering from a life-threatening disease. These findings suggest that although it is challenging to engage people in education about palliative care [52], active and participatory strategies as those implemented in this action-research study can be useful and have a positive impact. The societal relevance of palliative care was also highlighted and the participants considered the education session and program as being revolutionary, excellent, exciting and enlightening.

### Strengths, limitations and further research

A major strength of our study is its originality and relevance to a wide audience. In fact, although specifically focused on a group of teenagers, our action-research study allowed the implementation of an education program about palliative care to parents and other citizens of the local community. Furthermore, our study used an action-research approach, which is considered both to be a relevant approach in palliative care research and in health education and promotion [27–29, 31–34, 53]. The inclusion of a wide range of instruments and techniques for data collection ensures the validity and reliability of our findings [40–49]. Nevertheless, a few limitations need to be considered. First, as the education program was developed in a parish, some religious bias needs to be reflected and findings cannot be widespread to other settings. Second, as this is a single-centre study, some caution is needed in the interpretation and generalization of the findings. Further research (including the replication of this study on other contexts) and other education-program specifically focused on teenagers and local communities, and using other educational approaches (e.g., web-based ones) [54] need therefore to be promoted. For instance, health education programs about pressing issues, such as palliative and end-of-life care, and using multi-centred designed interventions could be designed as part of high school education, requiring further attention and study.

## Conclusions

The education program about palliative care was deemed to be excellent, exciting and enlightening by the teenagers to whom it was specifically designed. Positive feedback was given by the teenagers, parents and other participants. The findings of this action-research study show that the education program and education-intervention contributed to create awareness about palliative care among the participant teenagers, who ended the program with a positive message about palliative care.

### Implications of this study

The societal challenges of ageing populations have been recognized worldwide. Moreover, the relevance of community involvement and the active participation of citizens are seen as making a valuable contribution to the development of palliative care [17]. Nevertheless, although attention to palliative care is increasing, further developments and policy initiatives are needed to improve access to and quality of palliative care for all citizens who are in need of this type of care. The following policy implications can be drawn based on our findings. First, while our study highlights the positive impact of education initiatives about palliative care in early ages, further research is needed to assess the actual impact of education about palliative care in the acquisition of specific knowledge, development of competences and change of attitudes among teenagers and younger age groups (e.g., children). Second, education about palliative and end of life care should be promoted at local communities, for instance in primary and secondary schools, to foster community involvement, participation and empowerment. Finally, compassionate communities, described as networks that could encourage people to take some active responsibility for care and recognize that ageing and dying, death and bereavement are part of everyday life and happen to everyone [18–20], could and should be promoted to enhance the health and wellbeing of all citizens at the end of their life.

## References

[1] Zimmerman EB, Woolf SH, Haley A, Kaplan R, Spittel M, David D (2015). Understanding the relationship between education and health: a review of the evidence and an examination of community perspectives. Population health: behavioral and social science insights. AHRQ publication no. 15–0002. Rockville: Agency for Healthcare Research and Quality and Office of Behavioral and Social Sciences Research, National Institutes of Health.

[2] Wallerstein N, Bernstein E (1988). Empowerment education: Freire's ideas adapted to health education. Health Educ Q.

[3] Thompson B, Molina Y, Viswanath K, Warnecke R, Prelip ML (2016). Strategies to empower communities to reduce health disparities. Health affairs (Project Hope).

[4] Kumar S, Preetha G (2012). Health promotion: an effective tool for Global Health. Indian J Community Med.

[5] Council of Europe. Recommendation rec (2003) 24 of the Committee of Ministers to member states on the organisation of. palliative care. 2003;

[6] World Health Organization. Strengthening of palliative care as a component of integrated treatment throughout the life course. EB134/28. 134th session; 2013. 10.3109/15360288.2014.911801 24779434

[7] Davies E, Higginson IJ. Better palliative care for older people. Copenhagen: World Health Organization; 2004.

[8] Hall S, Petkova H, Tsouros AD, Costantini M, Higginson IJ. Palliative care for older people: better practices. Copenhagen: World Health Organization; 2011.

[9] Kite S (2006). Palliative care for older people. Age Ageing.

[10] Bone AE, Gomes B, Etkind SN, Verne J, Murtagh FE, Evans CJ, Higginson IJ (2018). What is the impact of population ageing on the future provision of end-of-life care? Population-based projections of place of death. Palliat Med.

[11] Currow DC, Phillips J, Agar M (2017). Population-based models of planning for palliative care in older people. Curr Opin Support Palliat Care.

[12] McIlfatrick S, Noble H, McCorry N (2013). Exploring public awareness and perceptions of palliative care. Palliat Medicine.

[13] McIlfatrick S, Hasson F, McLaughlin D (2013). Public awareness and attitudes toward palliative care in Northern Ireland. BMC Palliat Care.

[14] Benini F, Fabris M, Pace DS (2011). Awareness, understanding and attitudes of Italians regarding palliative care. Ann Ist Super Sanità.

[15] Stjernswärd J, Foley K, Ferris FD (2007). The public health strategy for palliative care. J Pain Symptom Manag.

[16] Pillemer K, Chen EK, Riffin C, Prigerson H, Reid MC (2015). Practice-based research priorities for palliative care: results from a research-to-practice consensus workshop. Am J Public Health.

[17] Martins Pereira S, Albers G, Pasman R, Van den Block L, Albers G, Martins Pereira S (2015). A public health approach to improving palliative care for older people. Palliative care for older people. A public health perspective.

[18] Kellehear A, Mitchell G (2008). Health promotion and palliative care. *Palliative Care: A Patient-Centred Approach*. Oxford: Radcliffe publishing.

[19] Kellehear A, Van den Block L, Albers G, Martins Pereira S (2015). Compassionate communities: caring for older people towards the end of life. Palliative care for older people. A public health perspective.

[20] Kellehear A (2013). Compassionate communities: end-of-life care as everyone’s responsibility. Q J Med.

[21] Wilkinson R, Marmot M, editors. *Social Determinants of Health. The solid facts*. Denmark: World Health Organization; 2003.

[22] Paul S, Cree VE, Murray SA. Integrating palliative care into the community: the role of hospices and schools. *BMJ Support Palliat Care*. 2017. First published on April. 2017:16. 10.1136/bmjspcare-2015-001092. 10.1136/bmjspcare-2015-001092 27515864

[23] Kelly MP, Morgan A, Bonnefoy J, et al. *The social determinants of health: Developing an evidence base for political action Final Report to World Health Organization, Commission on the Social Determinants of Health and the Measurement and Evidence Knowledge Network*. United Kingdom: National Institute for Health and Clinical Excellence and Chile: Universidad del Desarollo; 2007.

[24] Araújo J, Costa Gomes C, Jácomo A, Martins Pereira S (2017). Teaching bioethics in high schools. Health Educ J.

[25] Beccaro M, Gollo G, Giordano M (2014). The Ligurian high-school educational project on palliative care: development and piloting of a school-based intervention on bereavement and severe illness. Am J Hosp Palliat Med.

[26] Beccaro M, Gollo G, Ceccon S (2015). Students, severe illness, and palliative care: results from a pilot study on a school-based intervention. Am J Hosp Palliat Med.

[27] Ivankova NV (2015). Mixed methods applications in action research. From methods to community action.

[28] James EA, Milenkiewicz MT, Bucknam A (2008). Participatory action research for educational leadership. Using data-driven decision making to improve schools.

[29] Reason P, Bradbury H (2008). Handbook of action research: participative inquiry and practice.

[30] Waterman H, Saks M, Allsop J (2013). Action research and health. Researching health. Qualitative, quantitative and mixed methods.

[31] Sealey M, O'Connor M, Aoun SM, Breen LJ (2015). Exploring barriers to assessment of bereavement risk in palliative care: perspectives of key stakeholders. BMC Palliat Care..

[32] Hockley J, Froggatt K, Heimerl K (2012). Palliative care and participatory research: actions and reflections.

[33] Froggatt K, Hockley J (2011). Action research in palliative care: defining an evaluation methodology. Palliat Med.

[34] Cooper J, Hewison A (2002). Implementing audit in palliative care: an action research approach. J Adv Nurs.

[35] Sandoval JUA, Lucero J, Oetzel J (2012). Process and outcome constructs for evaluating community-based participatory research projects: a matrix of existing measures. Health Educ Res.

[36] Brown DR, Hernández A, Saint-Jean G, Evans S, Tafari I, Brewster LG, Celestin MJ, Gómez-Estefan C, Regalado F, Akal S, Nierenberg B, Kauschinger ED, Schwartz R, Page JB (2008). A participatory action research pilot study of urban health disparities using rapid assessment response and evaluation. Am J Public Health.

[37] Seymour J, Almack K, Kennedy S (2010). Implementing advance care planning: a qualitative study of community nurses' views and experiences. BMC Palliat Care..

[38] van de Geer J, Veeger N, Groot M, Zock H, Leget C, Prins J, Vissers K. Multidisciplinary training on spiritual Care for Patients in palliative care trajectories improves the attitudes and competencies of hospital medical staff. Am J Hosp Palliat Care. 2017; 10.1177/1049909117692959. 10.1177/1049909117692959 28193104

[39] Learmonth AM (2000). Utilizing research in practice and generating evidence from practice. Health Educ Res.

[40] Carr W, Kemmis S. *Becoming critical: Education, knowledge and action research.* Geelong: Deakin: University Press; 1986.

[41] Germain A, Nolan K, Doyle R, et al. The use of reflective diaries in end of life training programmes: a study exploring the impact of self-reflection on the participants in a volunteer training programme. BMC Palliat Care. 2015;15(28) 10.1186/s12904-016-0096-5. 10.1186/s12904-016-0096-5 PMC4779245 26944056

[42] Vaismoradi M, Turunen H, Bondas T (2013). Content analysis and thematic analysis: implications for conducting a qualitative descriptive study. Nurs Health Sci.

[43] Mertens DM, Hesse-Biber S (2012). Triangulation and mixed methods research: provocative positions. Journal of Mixed Methods Research.

[44] Creswell J (2003). Research design: qualitative, quantitative and mixed methods approaches.

[45] Golafshani N (2003). Understanding reliability and validity in qualitative research. Qual Rep.

[46] Shenton AK (2004). Strategies for ensuring trustworthiness in qualitative research projects. Educ Inform.

[47] Zohrabi M (2013). Mixed method research: instruments, validity, reliability and reporting findings. TPLC.

[48] Noble H, Smith J (2015). Issues of validity and reliability in qualitative research. Evid Based Nurs.

[49] Leung L (2015). Validity, reliability, and generalizability in qualitative research. J Family Med Prim Care.

[50] Pousset G, Bilsen J, De Wilde J (2009). Attitudes of Flemish secondary school students towards euthanasia and other end-of-life decisions in minors. Child Care Health Dev.

[51] Boucher NA (2016). Direct engagement with communities and Interprofessional learning to factor culture into end-of-life health care delivery. Am J Public Health.

[52] O’Connor M, Abbott J-A, Recoche K (2012). Getting the message across: does the use of drama aid education in palliative care?. Adv Health Sci Educ.

[53] Marsh P, Gartrell G, Egg G (2017). End-of-life care in a community garden: findings from a participatory action research project in regional Australia. Health Place.

[54] Paul CL, Carey ML, Sanson-Fisher RW (2013). The impact of web-based approaches on psychosocial health in chronic physical and mental health conditions. Health Educ Res.

